# Testing a Generalizable Machine Learning Workflow for Aquatic Invasive Species on Rainbow Trout (*Oncorhynchus mykiss*) in Northwest Montana

**DOI:** 10.3389/fdata.2021.734990

**Published:** 2021-10-18

**Authors:** S. Carter, C. B. van Rees, B. K. Hand, C. C. Muhlfeld, G. Luikart, J. S. Kimball

**Affiliations:** ^1^ Numerical Terradynamic Simulation Group, WA Franke College of Forestry and Conservation, University of Montana, Missoula, MT, United States; ^2^ Flathead Lake Biological Station, Division of Biological Sciences, University of Montana, Polson, MT, United States; ^3^ U.S. Geological Survey, Northern Rocky Mountain Science Center, Glacier National Park, West Glacier, MT, United States; ^4^ Department of Ecosystem and Conservation Sciences, WA Franke College of Forestry and Conservation, University of Montana, Missoula, MT, United States

**Keywords:** invasive species, machine learning, species distribution modeling, remote sensing, big data analytics, early detection and rapid response

## Abstract

Biological invasions are accelerating worldwide, causing major ecological and economic impacts in aquatic ecosystems. The urgent decision-making needs of invasive species managers can be better met by the integration of biodiversity big data with large-domain models and data-driven products. Remotely sensed data products can be combined with existing invasive species occurrence data via machine learning models to provide the proactive spatial risk analysis necessary for implementing coordinated and agile management paradigms across large scales. We present a workflow that generates rapid spatial risk assessments on aquatic invasive species using occurrence data, spatially explicit environmental data, and an ensemble approach to species distribution modeling using five machine learning algorithms. For proof of concept and validation, we tested this workflow using extensive spatial and temporal hybridization and occurrence data from a well-studied, ongoing, and climate-driven species invasion in the upper Flathead River system in northwestern Montana, USA. Rainbow Trout (RBT; *Oncorhynchus mykiss*), an introduced species in the Flathead River basin, compete and readily hybridize with native Westslope Cutthroat Trout (WCT; *O. clarkii lewisii*), and the spread of RBT individuals and their alleles has been tracked for decades. We used remotely sensed and other geospatial data as key environmental predictors for projecting resultant habitat suitability to geographic space. The ensemble modeling technique yielded high accuracy predictions relative to 30-fold cross-validated datasets (87% 30-fold cross-validated accuracy score). Both top predictors and model performance relative to these predictors matched current understanding of the drivers of RBT invasion and habitat suitability, indicating that temperature is a major factor influencing the spread of invasive RBT and hybridization with native WCT. The congruence between more time-consuming modeling approaches and our rapid machine-learning approach suggest that this workflow could be applied more broadly to provide data-driven management information for early detection of potential invaders.

## Introduction

Non-native, Invasive Species (IS) are causing severe biological and economic disruption worldwide (Sepulveda et al., 2012; Shackleton et al., 2019). IS are the second most prevalent driver of species extinctions (Bellard et al., 2016), with estimated financial damages amounting to over a hundred billion dollars annually in certain individual countries (Pimentel 2002; Bradshaw et al., 2016). Continued anthropogenic landscape change and climate change may favor invaders by shifting competitive relationships with native species (Hellmann et al., 2008). Aquatic IS represent a particular threat to freshwater ecosystems due to their high potential for establishment and spread and severe ecosystem impacts (Havel et al., 2015). The current and predominant paradigm for IS management is Early Detection and Rapid Response (EDRR), but the intensive resources and surveillance involved in this framework’s implementation may be prohibitive without new and innovative uses of technology (Martinez et al., 2020). EDRR depends on frequent, widespread, and ongoing monitoring to enable timely response, but such monitoring is extremely labor intensive and likely beyond the capabilities of many management actors. Timely risk assessments allow for the spatial prioritization of monitoring that could streamline EDRR and its ability to prevent irreversible damage (Reaser et al., 2020a; Martinez et al., 2020), such that decision makers can focus surveillance and intervention efforts where they are likely to be most effective under budgetary and resource constraints. Such prioritizations are often based on heuristic preconceptions rather than data-driven approaches, and as such are neither repeatable nor transparent for system stakeholders. By contrast, scientifically informed, formal target screening may lack adequate temporal agility and accurate risk assessments. Many conventional modeling approaches to knowledge creation operate on long time scales (months to years) which may not be helpful to managers. Indeed, current modeling methodologies fail to provide managers with sufficient decision-making information in near real time (Bayliss et al., 2013).

Given the finite supply of resources and quick timelines for IS management, there is a need for improved expediency and accuracy in identifying areas of highest vulnerability to IS establishment.

Species Distribution Models (SDMs) have been widely applied as spatial decision support tools for IS managers (Srivastava et al., 2019) and can be broadly categorized into mechanistic and correlative model classes (Elith, 2017). Process-based, or mechanistic, models require considerable developmental and computational effort (Kearney and Porter, 2009) and can thus be out of sync with the needs for timely analyses for EDRR (Merow et al., 2011). These models rely on exhaustive, experimentally derived functional characteristics (Shabani et al., 2016) or hierarchal frameworks that are built to elucidate or test hypotheses about ecological relationships rather than simply predict patterns in species occurrence (see Muhlfeld et al., 2014; Berthon 2015; Muhlfeld et al., 2017; Farley et al., 2018).

On the other hand, correlative SDMs require less mechanistic understanding and instead rely on apparent relationships between species and environmental characteristics. Such models are comparatively quick to train and develop but are often built using low-resolution spatially interpolated climatic data, such as WorldClim (Hijmans et al., 2005; Elith et al., 2010; Fourcade et al., 2014). Since the WorldClim data (Fick and Hijmans, 2017) are not temporally explicit, and static covariates, by definition, cannot adequately provide a temporally continuous evaluation of risk, the value of these data for EDRR is hampered. Although a major drawback of these correlative models is that long-term extrapolation is more difficult, this disadvantage is outweighed by the acute need for rapid risk assessments to inform IS monitoring and biosurveillance. Indeed, facilitating IS management within the EDRR framework would be significantly improved by new workflows that can identify readily available drivers of invasion and establish relative invasion risk within the operational time scales of managers.

Many of the challenges outlined above can be met by data-driven and iterative workflows made possible by machine learning (ML) and the big data revolution (Runting et al., 2020). For instance, one challenge is the need for scalable and fast modeling workflows to guide managers and decision makers (Reaser et al., 2020a). ML algorithms are an increasingly viable method for many modeling problems involving big data, particularly when the primary objective is to achieve high levels of predictive accuracy rather than develop a mechanistic understanding of the study system (Bhattacharya, 2013). ML algorithms, particularly non-parametric iterative algorithms (e.g., random forests), are free from many strict assumptions such as independent observations and the need to avoid collinearity (Olden et al., 2008; Thessen 2016). In addition, ML models are well suited to the iterative modeling framework due to their automated approach, fast development process (Tarca et al., 2007) and highly scalable nature (Farley et al., 2018). This enables them to take advantage of other big data attributes, including its widespread proliferation, global coverage, and rapid updating (Whitehead et al., 2020). As new data become available, ML frameworks can be updated to reflect new understanding.

However, ML models are not a panacea: because they are immensely complex and, with the exception of intricate Bayesian ML models, do not incorporate the underlying uncertainty of the data (Cressie et al., 2009), making inferences about underlying processes less straightforward and dependent on the type of model being used (Farley et al., 2018; Parr et al., 2020). Nevertheless, the rapid, iterative, and predictive characteristics of ML approaches are an excellent match for the analytical needs of EDRR implementation, which prioritize speed and adaptiveness over mechanistic understanding.

Another challenge of EDRR is the availability and distribution of environmental data typically used to assess relative habitat suitability (Randin et al., 2020). Conventional spatially interpolated climate data often require enormous developmental effort (Daly et al., 2000; Hijmans et al., 2005), which, when temporally explicit, can hinder their utility in developing models that meet the adaptive (e.g., annually repeating) demands of EDRR. Moreover, because they are based on interpolations from global weather stations, such products yield high model uncertainty in areas with sparse geographic coverage (Bedia et al., 2013).

In contrast, Remote Sensing (RS) products available from global polar-orbiting environmental satellites have regular revisit intervals ranging from 1 to 16 days and are derived from spatially explicit observations, so the burden of geographic uncertainty is mitigated. Indeed, because of the complimentary nature and spatial and temporal continuity of many operational satellite records, RS observational data are expected to shape the next generation of SDMs (He et al., 2015) and are the preferred or perhaps the only option for regional, continental, and global scale prediction of IS spread (Leitão and Santos, 2019; Vaz et al., 2019). These products are sensitive to many environmental properties, such as surface temperature, that constrain and explain species occurrences (Randin et al., 2020). These and other satellite-based measurements have rarely been applied to SDMs relative to spatially interpolated climate data products (Dittrich et al., 2019), and their use for assessing species distributions has been increasing in recent years (Lausch et al., 2016; Randin et al., 2020).

Although the spatial and temporal continuity of RS data improves the transferability and precision of capturing ecological niche requirements in many terrestrial environments (Randin et al., 2020), stream environments represent a particular challenge in integrating technological advances with IS management. Because the 2-dimensional footprint of RS products is often larger than the footprint of streams, such products can only provide proxies for physiologically relevant conditions within the aquatic environment. Thus, models trained to link species occurrences with environmental remotely sensed information may fail to capture the actual processes experienced by aquatic organisms, and care must be taken to avoid spurious conclusions. Coherent workflows that link remote sensing data and machine learning functionalities are especially needed for freshwater systems to mobilize myriad spatial products in data-driven aquatic IS risk analysis.

Here, we demonstrate one such workflow linking these technologies to produce rapid and adaptable species distribution modeling for spatial risk assessments of aquatic IS. To provide proof of concept, we implemented this workflow on a well-documented case study of a climate-assisted species invasion. This worked case study allowed us to assess not only the predictive accuracy of this approach but also whether it gives meaningful insights into the environmental drivers of habitat suitability for a focal IS. Our study objectives were to: 1) Identify the most effective remotely sensed proxies for characterizing habitat suitability (a proxy for invasion risk) for our focal IS (RBT; *Oncorhynchus mykiss*); 2) Construct habitat suitability maps for spatial risk assessments using a combination of RS data products and ML methods; and 3) Test the feasibility of ML models for iterative reassessment of IS risk screening efforts within the EDRR framework.

### Study System

**FIGURE 1:**
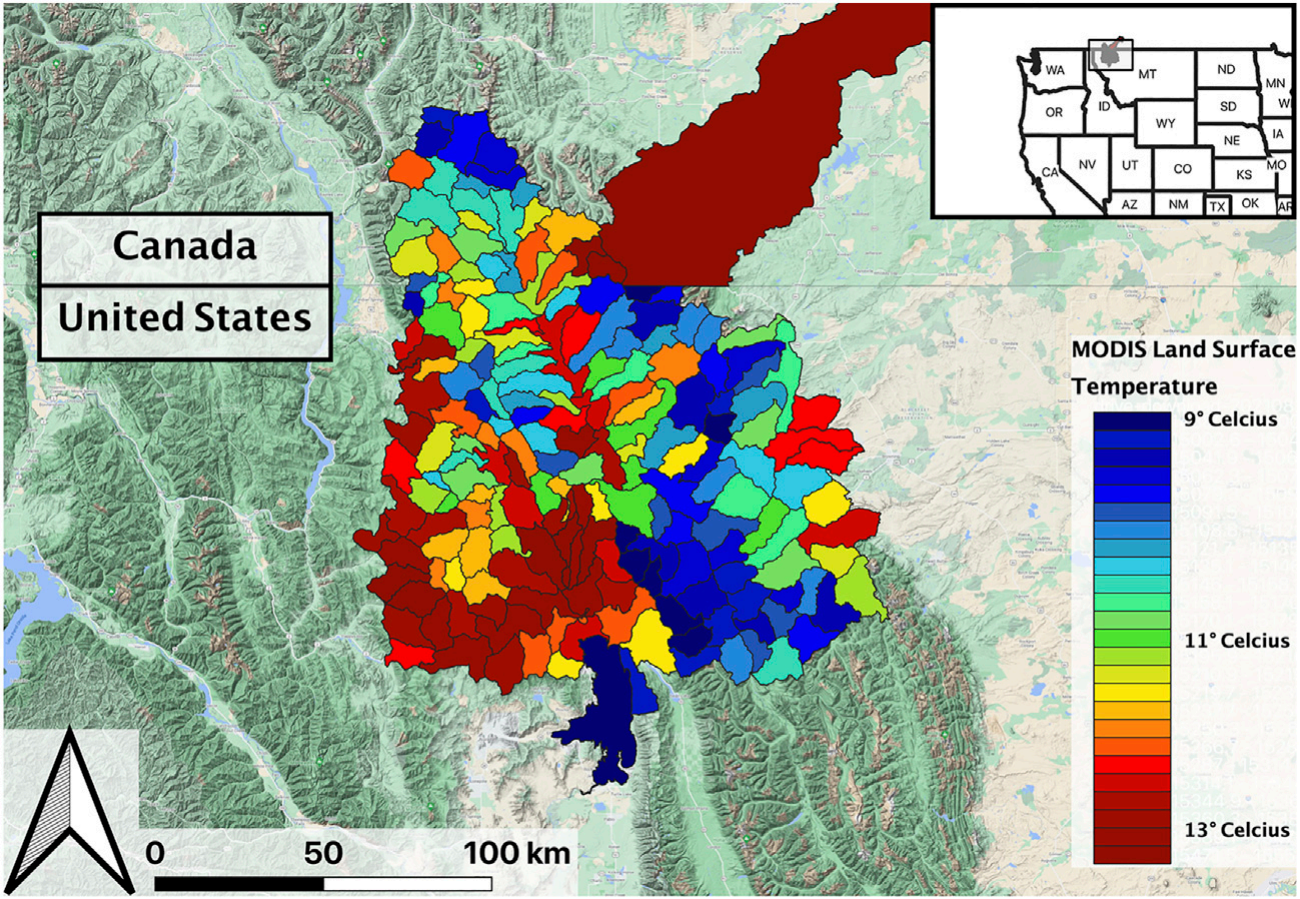
Overview of study area, including a sample data product (Land Surface Temperature) aggregated by hydrologic units.

The study area encompassed the tributaries of upper Flathead River system extending over portions of northwestern Montana United States, and southern British Columbia and Alberta, Canada (Figure 1). These mountain streams flow through forested landscapes and host several native fish species including Westslope Cutthroat Trout (WCT; *Oncorhynchus clarki lewisi*). Stream temperature and the timing and duration of peak streamflow events are key ecological drivers in these streams (Hauer et al., 2007), while the timing and intensity of snowmelt is a key driver influencing spring runoff in this system (Pederson et al., 2010; Wu et al., 2012).

Rainbow trout (*O. mykiss*) were artificially propagated and introduced into watersheds across the Continental United States for recreational purposes between 1870 and 1971 (Pister 2001; Bennett et al., 2010). Since their introduction into the Flathead River in 1880 (Hitt et al., 2003), RBT have been hybridizing with native WCT (Hitt et al., 2003; Allendorf et al., 2004; Boyer et al., 2008; Muhlfeld et al., 2017). The impacts of RBT on WCT populations, particularly due to the spread of RBT individuals and their alleles, has been tracked for decades (Kovach et al., 2016). The spread of alleles appears to be driven more by legacy introductions, and thus propagule pressure, than environmental conditions (Muhlfeld et al., 2017; Boyer et al., 2008). Relative to WCT, RBT prefer warmer temperatures, lower spring flows, earlier spring runoff, and tolerate greater environmental disturbance (Fausch et al., 2001; Muhlfeld et al., 2009a; Muhlfeld et al., 2009b; Bear et al., 2007). During spawning, WCT generally migrate greater distances and spawn during peak flows, whereas RBT spawn earlier (i.e., during periods of lower flows) and lower in the river system (Muhlfeld et al., 2009b). High flows can affect both RBT and WCT, although reduced spring flows and warmer water temperatures have been associated with increased spread of RBT hybridization in the Flathead River and across the northern Rocky Mountains (Muhlfeld et al., 2014; Muhlfeld et al., 2017), which are strongly influenced by spring precipitation, winter snowpack, and the timing of spring snowmelt (Pederson et al., 2010).

### Data Acquisition-Genetic and Genomic Data

Trout have been periodically captured, sampled, and genotyped to assess the degree of RBT genetic admixture (the proportion of RBT alleles at the population level) in the study system since 2000. We used the associated long-term genetic monitoring data between years 2002 and 2019 as an index of RBT invasion. U.S. Geological Survey and Montana Fish Wildlife and Parks personnel selectively sampled streams where there was concern that WCT were hybridizing with non-native RBT, collecting fin clips from electrofished individuals and genotyping these individuals using various markers (microsatellites, SNPs, RAD-Capture sequencing). The genetic data were used to calculate RBT admixture in sampled populations.

### Data Acquisition-Presence Absence Data

We generated a presence-absence dataset by classifying all occurrence records of less than 10% admixture to be “absent.” Although 10% still represents the presence of RBT alleles, conditions at these locations are less favorable for the establishment of this invasive taxon. Considering the difficulty of acquiring actual absence data (Jiménez-Valverde et al., 2008) and that many SDM’s rely on “pseudo absences”–background points used to characterize the range of environmental conditions in a given study area (Lobo et al., 2010)–we assume that these genotypic absences contain insightful information regarding the distribution of RBT, particularly in comparison to pseudo absences. We supplemented these absences with a RBT dataset acquired from the Non-indigenous Aquatic Species (NAS; U.S. Geological Survey, 2020) database and clipped these records to the bounding box of the RBT genetics dataset. We included only data records acquired after year 2002 to match the availability of RS data. We also corrected for the influence of spatial autocorrelation by systematically subsampling data records so that no two points fell within 500 m of each other in a given year (Fourcade et al., 2014). The resultant occurrence dataset included 323 RBT presence locations and 167 absence point locations distributed across the study region over a 14 year record. The occurrence data were then joined to Hydrologic Unit Catchment polygons (HUC; Seaber et al., 1987). HUC polygons represent the landscape catchment area that drains to a portion of the stream network, whose hierarchical structure allows for a multi-scale delineation of drainage systems.

### Data Acquisition and Processing-RS Data

To test whether proximal remote sensing cues contain sufficient environmental information to capture RBT niche requirements, we selected a number of readily available satellite RS data products based on a priori assumptions of ecologically relevant drivers of hybridization and distribution (see below; Table 1). To avoid scale mismatch issues among predictors, we modeled environmental variables aggregated over HUC-12 polygons at the sub-watershed scale. Aggregating each covariate to HUC polygons mitigates the potential footprint mismatch between the RS observations and stream network within a catchment and is a common technique used in building freshwater SDMs in order to handle issues of scale relating to predictor variables (Friedrichs-Manthey et al., 2020). In addition, this method alleviates the inconsistent sampling inherent in the data and implicitly accommodates the mobile nature of RBT. Here, we give a brief description of the data products selected for model training and their connection to RBT niche requirements. The data products were preprocessed before being spatially aggregated to HUC-12 polygons as follows.

**TABLE 1:** Library of hypothesized and known ecologically relevant drivers of RBT hybridization and distribution.

Environmental Covariate	Source	Description	Hypothesized Ecological Connection	Units	Resolution
Land Surface Temperature	MODIS AQUA LST MYD11A2 (V6; Wan et al., 2015)	Temperature on the surface of the Earth measured using thermal infrared passive sensors	Stream Temperature; Maximum annual temperature record	Kelvin	1 km
Precipitation^a^	National Land Data Assimilation System (NLDAS; Mitchell 2004)	Rain and snow accumulation, interpolated from weather stations and integrated with actively sensed radar products	Magnitude of peak flow events	kg/m^2^	0.125 arc degrees 10 km
Flashiness^a^	USGS Dynamic Surface Water Extent Product (Jones 2018)	Annual per-pixel variation of a dynamic surface water extent algorithm; Derived from Landsat satellite imagery	Flood disturbances; Seasonal flow variation	Unitless	30 m
Surface Water Occurrence	JRC Global Surface Water Mapping Layers (Pekel et al. 2016)	Persistence of water on the surface; Derived from Landsat satellite imagery	Stream flow rates (at HUC - level aggregation); Habitat connectivity	Unitless	30 m
Topographic Diversity	Theobald et al. (2015)	Variation in temperature and moisture conditions available to species	Habitat structure and diversity	Unitless	90 m
Gross Primary Productivity	Robinson et al. (2018)	Amount of carbon captured by plants in an ecosystem; Derived from Landsat satellite imagery	Carbon available to the system	kg C/m^2^/16-days	30 m
Normalized Difference Vegetation Index	MODIS AQUA MYD13A2 (V6) Vegetation Indices	Density of “greenness” on landscape	Photosynthetic Activity	Unitless	250 m
Enhanced Vegetation Index	MODIS AQUA MYD13A2 (V6) Vegetation Indices	Modified vegetation index that reduces atmospheric contamination and maintains sensitivity over dense vegetation	Photosynthetic Activity relative to Canopy Structure	Unitless	250 m
Percent Tree Cover	MODIS TERRA MOD44B (Hansen et al., 2003)	Percent of woody vegetation	Stream structure and habitat diversity	Percent cover	250 m
Heat Insolation Load	Theobald et al. (2015)	Incident radiation derived from latitude, slope, and aspect	Daily temperature variation; Stream Temperature	Unitless	90 m

a Preprocessed further from published products (see methods).

Land Surface “skin” Temperature (LST) observations were obtained from thermal-infrared measurements from the Moderate Resolution Imaging Spectroradiometer (MODIS) mounted on the NASA EOS Aqua satellite (Li et al., 2013; Wan et al., 2015). The MODIS LST product is mapped to a 1-km resolution spatial grid similar to the sensor footprint. LST retrievals are acquired on a daily basis and composited over coarser 8-day intervals to reduce cloud and atmosphere contamination effects. The MODIS Aqua LST retrievals are acquired at 13:30 local time from the sun-synchronous polar orbiting satellite and reflect mid-day conditions close to the maximum diurnal temperature range. Because trout species are limited by high temperature (Wenger et al., 2011), we constructed a maximum composite image by capturing the maximum LST recorded in each grid cell for each year in our study period.

The National Land Data Assimilation System (NLDAS) uses a land surface model to integrate ground and space based observing systems, providing spatially explicit and temporally continuous estimates for various environmental variables including precipitation, potential evaporation, and specific humidity (Mitchell, 2004) at 0.125 arc° and hourly resolutions. We aggregated the NLDAS precipitation product with a per-pixel sum composite at 3-month seasonal intervals (i.e., Spring Precipitation, Summer Precipitation, etc).

The Dynamic Surface Water Extent (DSWE) product provides high temporal (8-days) repeat, moderate spatial resolution (30 m) data on surface water inundation across broad spatial scales (Jones 2019). It uses an experimentally derived spectral mixture model and 5 rule-based decision criteria to classify Landsat surface reflectance pixels as “not water,” “open water,” or “partial surface water” in a spatially and temporally explicit manner. For each week in our study period (i.e., 2002–2018), we gathered DSWE observations and generated a weekly per-pixel estimate of surface water inundation in our study area. We produced a surface water variation metric by finding the per-pixel temporal standard deviation within each year. The temporal standard deviation (as opposed to the IQR or variance) of the water variation was chosen as a proximal cue for stream flashiness due to its sensitivity to outliers, since RBT spawning is known to be sensitive to variations in stream flow rates.

In contrast to the DSWE product, the Landsat global surface water extent product identifies the presence of water over time using a mix of expert systems, visual analytics, and evidential reasoning (Pekel et al., 2016). Using this algorithm, Pekel et al. (2016) developed several thematic mapping layers including the Surface Water Occurrence metric, which quantifies the overall location and persistence of surface water cover at 30 m spatial resolution from 1984 to present. The surface water persistence metrics are derived from the Landsat satellite series record, which provides consistent 30 m spatial resolution and potential 16-days repeat coverage over the globe. However, actual spatial and temporal coverage of surface water dynamics is degraded by cloud and atmosphere contamination, seasonal reductions in solar illumination at higher latitudes, and overlying vegetation cover. Slow moving main-stem rivers generally have larger surface areas than lower order streams, so when spatially aggregated to HUC-level polygons, this product encapsulates information about flow rates and overall aquatic habitat connectivity.

Gross Primary Productivity (GPP) quantifies the plant photosynthetic uptake of atmospheric CO_2_ and represents the amount of carbon and energy flow into the ecosystem. In this study, a 30 m resolution daily GPP record for the continental United States was used to characterize energy (and nutrients) available to ultimately support aquatic food webs. The GPP record is calculated using a modified form of the MOD17 light use efficiency algorithm driven by satellite observed fraction of photosynthetic active radiation (FPAR) derived from Landsat 30 m spectral reflectances, gridded (4-km resolution) daily surface meteorology observations (i.e., gridMET; Abatzoglou 2013), and the national land cover database (Robinson et al., 2018). GPP has been used to predict freshwater fish species richness across the globe (Pelayo-Villamil et al., 2015), and previous research supports the link between primary production and fish productivity (Downing et al., 1990). Thus, this proximal product may contain information pertaining to the invertebrate community or vegetation structure. We calculated the accumulated annual GPP during each year of interest as a temporal sum composite, hypothesizing that the Landsat based GPP record captures bioenergetic constraints at scales relevant to RBT.

The MODIS Enhanced Vegetation Index (EVI; Didan 2015) is a modified version of the Normalized Difference Vegetation Index (NDVI), has improved sensitivity to green vegetation cover in high biomass regions, and minimizes atmospheric contamination effects. The MODIS (MOD13Q1) EVI product is derived globally at 250m, 16-days spatiotemporal resolutions. Because plants both absorb radiation in the visible spectrum and emit radiation in the near-infrared spectrum, the EVI is sensitive to the photosynthetic activity of terrestrial systems. Massicotte et al. (2015) used EVI as a proxy for aquatic vegetation biomass to predict larval fish abundance. Here, we used EVI as a proxy for the potential productivity of stream and riparian systems, where higher productivity systems would be more susceptible to invasion (i.e., hot spots). Thus, we calculated a temporal EVI mean composite for each year to capture average conditions relevant to RBT.

The NASA MODIS Vegetation Continuous Fields (VCF) product provides a spatially continuous land cover estimate of general vegetation traits such as percent tree cover, percent non-tree cover, and percent barren land at 250 m resolution and annual temporal fidelity (Hansen et al., 2003). The MODIS (MOD44B) VCF product is derived using a decision tree classification trained on MODIS surface reflectance and LST. We used the VCF percent tree cover metric to define the vegetative structure of the system within each HUC. The vegetation structure of various riparian areas has been linked to macro-invertebrate species richness (Sweeney 1993; Death and Collier 2009). We chose the VCF product to represent the overall disturbance and shadiness of a given HUC. Although GPP, EVI, and Percent Tree Cover quantify similar aspects of bioenergetic constraints, macro-invertebrate potential, and habitat structure, we expected to see differences in predictive power due to their differing resolutions, underlying algorithms, and retrieval accuracy.

In addition, topographic indices such as Topographic Diversity and Heat Insolation Load (Theobald et al., 2015) provide information about the topographic structure, microclimate variability, and resultant thermal dynamics of a given HUC. Topographic diversity is also congruent with the measurement of the heterogeneity of various landforms including valley bottom constraints, hills, and ridges as derived from a multi-scale neighborhood analysis. This metric indicates the structural diversity and, therefore, the likelihood of connectivity of stream networks within watersheds. Heat Insolation Load reflects variations in latitude and incident solar radiation to quantify the heat-loading capacity of different regions. Together with LST, heat insolation load provides a proximal cue to the overall stream temperature of a given HUC.

Covariates were obtained through data preprocessing performed within Google Earth Engine (GEE; Gorelick et al., 2017). We subjected each lower-level remote sensing variable (e.g. LST, GPP, EVI, Percent Tree Cover) to stringent quality filtering based on pre-published quality bands included in each product (see Supplementary Material S1). We kept the quality control filters inherent in the higher-level development products (e.g. Surface Water Occurrence, Heat-insolation Load). We intersected the RBT survey locations to their encompassing HUC12 catchments and calculated a weighted average of genetic admixture relative to the number of individuals in a dataset. For the RBT occurrence dataset, we simply aggregated occurrence points to the HUC level. We classified any HUC containing at least one presence location to be suitable. We then averaged each environmental covariate across all HUCs in our study area. This resulted in a tabular dataset with each column corresponding to the spatial average of an environmental covariate, or—depending on what our dependent variable was—a HUC-level weighted admixture percentage or HUC-level occurrence Boolean. By taking HUC-level aggregates, we controlled for the effects of steep topography that concentrate environmental gradients at small spatial scales and the potential footprint mismatch between environmental data pixels and stream conditions. Although the same HUC may have been sampled in multiple years, we treated each HUC–year pair as an independent observation.

**FIGURE 2:**
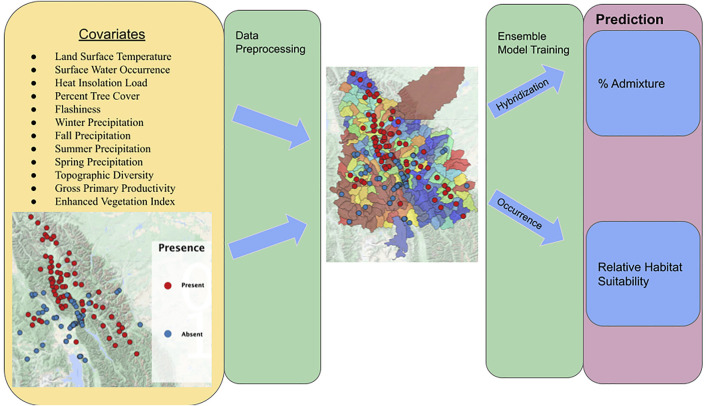
Overall workflow, model inputs, and model outputs. Yellow box indicates model inputs. Green boxes indicate steps as referenced in the methods. Purple box indicates each model output. RBT presence and absence observation locations are denoted by respective red and blue points on the associated study area maps.

Data were exported from GEE, and due to the reliance of variable importance techniques on predictors being independent of one another, all covariates with a Pearson’s correlation coefficient >0.7 were dropped (Dormann et al., 2013). In addition, because covariates may contain similar explanatory information but may not be represented by a linear relationship, we tested for multicollinearity (Mansfield and Helms 1982) by fitting Random Forest models with each covariate as an independent variable, and we dropped each variable that was shown to have a feature dependence score >0.7 in predicting another variable. This process was repeated until no two columns had a partial dependency exceeding 0.6. This process resulted in 12 covariates: land surface temperature, surface water occurrence, heat insolation load, percent tree cover, flashiness, winter precipitation, fall precipitation, topographic diversity, summer precipitation, spring precipitation, gross primary productivity, and enhanced vegetation index. An overview of model inputs, outputs, and overall workflow can be found in Figure 2.

### Admixture Model Training

Using the above covariates, we trained an ensemble of Linear Regression (GLM), Gradient Boosted Regressor (GBM), Classification Tree Regressor (CTA), Artificial Neural Network Regressor (ANN), XGBoost Regressor (XGB), and Random Forest Regressor (RF) models using sklearn version 0.23.1 (Pedregosa et al., 2011) in Python 3.7.7, with 20% of data randomly withheld for testing. We used the ensemble method because it has been shown to be an improvement over single models by reducing model-based uncertainty (Marmion et al., 2009; Elith et al., 2010). For a brief description of each component model, see Supplementary Material S2. Because the distribution of RBT hybridization was severely skewed toward higher rates (i.e., right skewed), we visually confirmed that testing data had similar distributions to training data. To consolidate model estimates, we implemented an ensemble method consisting of each of the above models, weighting the overall prediction by the mean absolute error (Willmott and Matsuura 2005) and omitting the artificial neural network due to severe inaccuracy.

### Presence Absence Model Training

The same covariates were used for both the hybridization and occurrence models. We implemented an ensemble method consisting of the classification analogues for the above regression models, again using Scikit-learn version 0.23.2. We took a weighted average of each component model prediction by the area under the receiver operative characteristic curve statistic (i.e., AUC score; Bradley 1997), omitting the GLM and ANN due to the unrealistic predictions (see below; Elith et al., 2010). For example, if the random forest model were to have a higher accuracy score than the decision tree model, the overall ensemble model prediction would be more influenced by the random forest than the decision tree. We evaluated the predictive accuracy of the resultant ensemble model by computing a 30-fold cross validation accuracy score, where the training data were partitioned into 30 random segments of equal size, 29 of which were used to train the model, while the remaining segment was used to calculate the accuracy score. We calculated this accuracy score by computing the fraction of correct predictions of each segment, averaging the scores over all 30 folds for an overall metric of ensemble model accuracy. We then generated choropleth range maps (i.e., thematic maps showing summary statistics over a set number of polygons) by applying the ensemble of models to predict suitable habitat for mean covariates across two vector datasets representing the “first decade” (years 2002–2010) and the “second decade” (2010–2018) of the study period, each spatially aggregated to HUC level. Although each ensemble model predicted different presence amounts for the testing dataset, both the GLM and ANN did not show any variation of predicted suitability among first decade and 2nd decade HUCs, so were removed from further analysis. To examine the degree of extrapolation, we calculated the Multivariate Environmental Similarity Surface (Elith et al., 2010) for each vector dataset. To examine the model prediction certainty, we calculated the standard deviation of prediction probabilities for each remaining estimator.

### Discerning Top Predictors

To identify top predictors of RBT distributions, we implemented an ensemble of different feature importance techniques with each of the aforementioned ML models trained to predict occurrence and their analogues trained to predict hybridization. Each model was subject to Recursive Feature Elimination (Chen et al., 2018), Permutation Importance (Altmann et al., 2010), and Backwards Elimination (Draper and Smith, 1981). These feature importance methods are similar, but contain some important distinctions. Recursive Feature Elimination iteratively drops features which have the smallest impact on model prediction until a pre-defined number of features is leftover. Permutation Importance iteratively shuffles the values of a given predictor, predicts using all covariates including the artificially permuted feature, and measures the subsequent drop in classification accuracy. The predictor whose permutation yields the largest drop in classification accuracy is identified as the most important predictor. Backwards Selection drops a single predictor entirely, retraining a different model for each iteration and again measuring the drop in predictive performance. The top three predictors were selected for each remaining model and importance technique, and we tallied the number of times a given predictor was found in the top three. We also interrogated partial dependency plots for known mechanisms driving occurrence and hybridization.

## Results

The tree-based methods (i.e., Random Forest, Decision Tree, Gradient Boosted Trees, XGBoost) yielded higher predictive accuracy than the linear and deep learning models for the RBT application (Table 2). Although the occurrence ANN and logistic regression models predicted a mix of RBT presence and absence for an unseen test dataset, both models predicted homogenous vectors of presence or absence for the first and second decades. For instance, the logistic regression predicted that all HUCs in both decades were suitable; conversely, the ANN predicted that all HUCs in both decades were unsuitable. Similarly, both the hybridization ANN and linear regression models predicted unrealistic hybridization levels of 100% for every HUC, whereas all the tree-based regressors predicted RBT hybridization levels between 0 and 100%.

**TABLE 2:** Predictive capability of each ensemble model. Bold indicates highest accuracy models. Asterisk indicates models that were removed due to unrealistic predictions.

Occurrence	Model	Area Under the Curve Score
	**Random Forest**	**0.89**
	Logistic Regression	0.69
	Artificial Neural Network *	0.62
	Gradient Boosted Trees	0.84
	XGBoost	0.83
	Classification Tree	0.81
**Hybridization**	**Model**	**Mean Absolute Error**
	**Random Forest**	**0.05**
	Linear Regression	0.07
	Artificial Neural Network *	121.79
	**Gradient Boosted Trees**	**0.05**
	XGBoost	0.06
	**Classification Tree**	**0.05**

**FIGURE 3:**
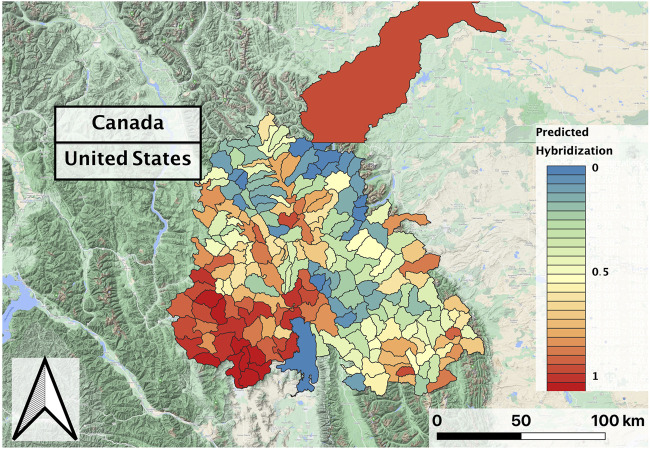
Predicted RBT hybridization for the second decade (2010–2018) composite, with dimensionless hybridization levels ranging from low (0) to high (1); black lines delineate individual HUCs within the larger study basin.

In evaluating the hybridization predictor (i.e., the ensemble of regression models), Land Surface Temperature, Heat Insolation Load, and Gross Primary Productivity were the most predictive features explaining RBT hybridization trends. The ensemble model also produced a favorable Mean Absolute Error of 5.5%. 90% of the residuals were less than 15% hybridization, although some predicted hybridization values had errors greater than 15%. Although observed hybridization percentages ranged from 0 to 100%, admixture predictions only ranged from 0 to 60%. Choropleth maps trained on the hybridization dataset did not correspond with known hybridization levels within the study area and showed unrealistic spatial patterning (i.e., checkerboarding rather than being spatially correlated) (Figure 3).

**FIGURE 4:**
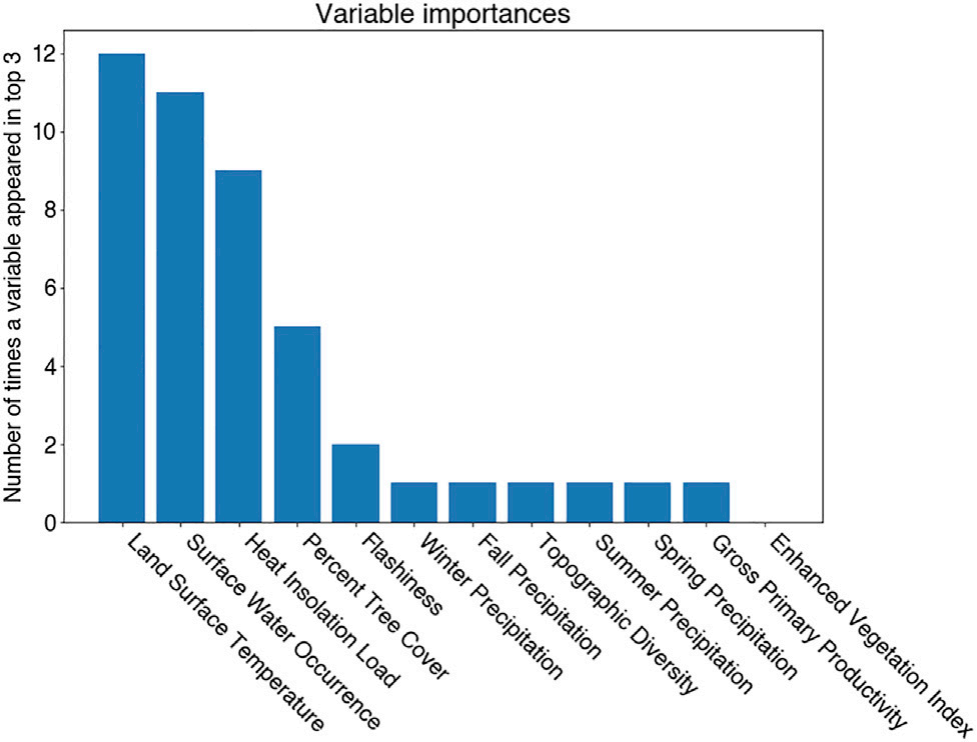
Top predictors of RBT occurrence as identified by the occurrence model.

**FIGURE 5:**
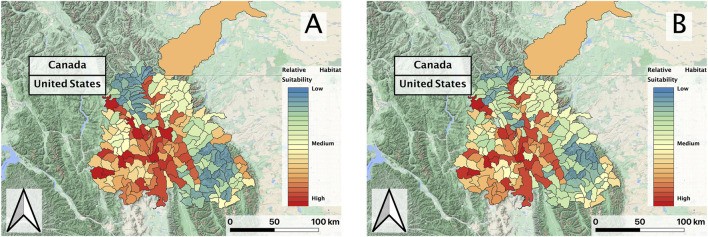
**(A)** Predicted RBT relative suitability of first decade (2002–2010) and **(B)** second decade (2010–2018) vector composites within the Flathead basin study region; black lines delineate individual HUCs within the larger basin.

**FIGURE 6:**
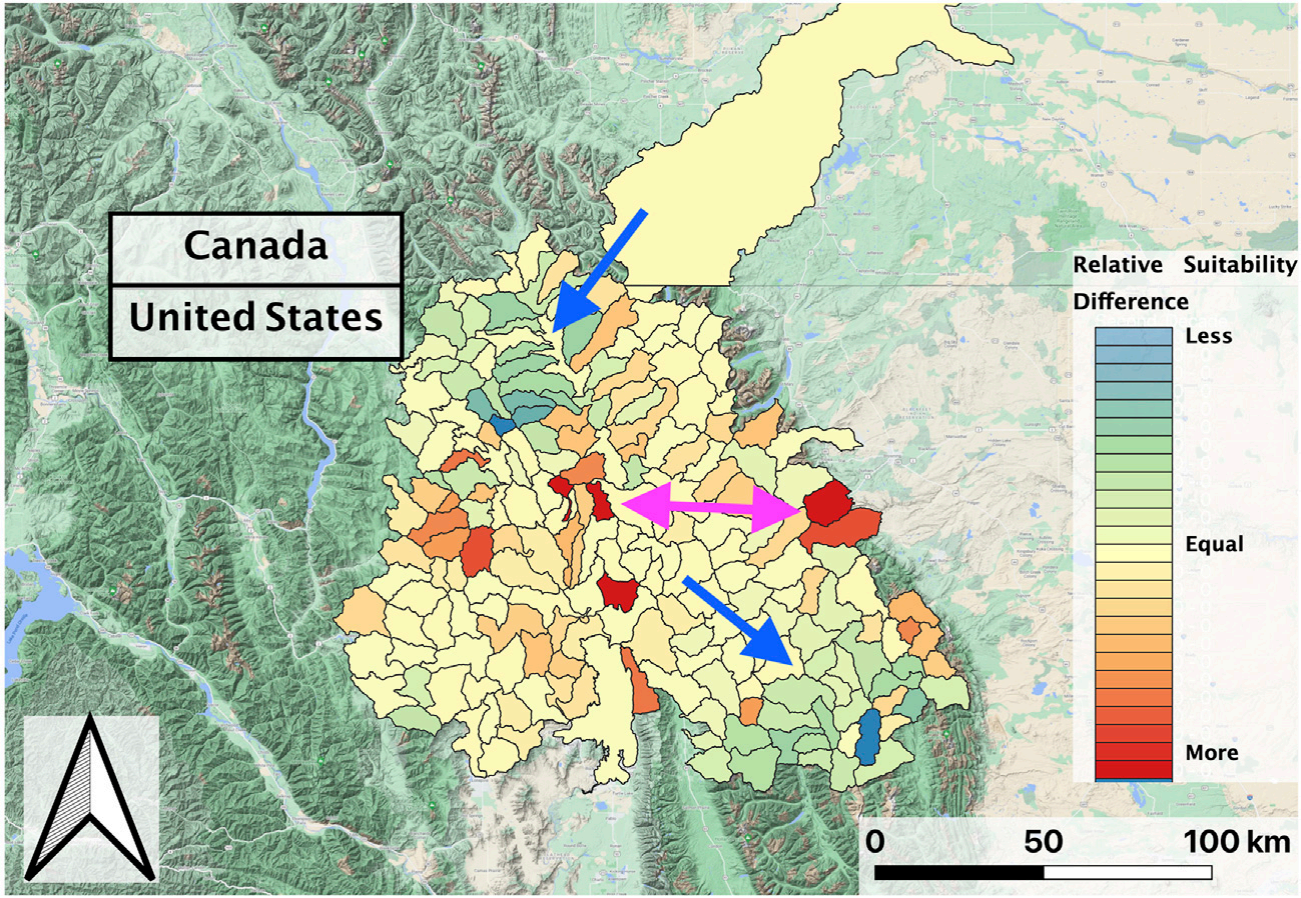
Normalized predicted relative RBT suitability change between the second and first decades of the study period (2002–2018) within the Flathead basin. The Salish Mountains and Lewis Range sub-regions decreased in suitability (blue-green shades; blue arrow), while suitability marginally increased in other regions and increased more drastically in portions of the northern Mission Range and east Glacier National Park regions (red shades; pink arrow).

**FIGURE 7:**
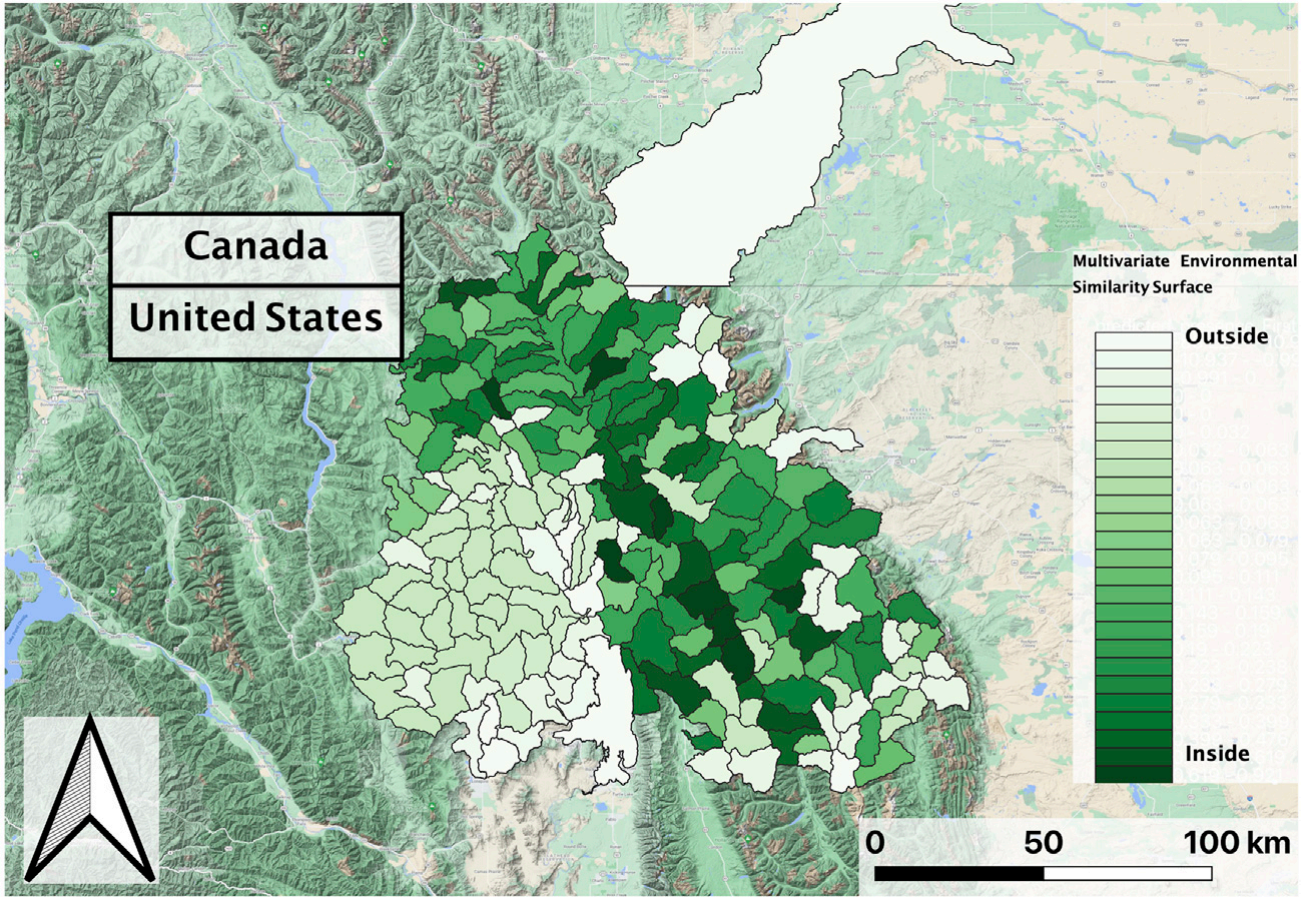
Multivariate Environmental Similarity Surface in the Flathead basin for the 2nd decade (2010–2018) vector composite, which was consistent with the first decade (2002–2010) composite. Greener shades in the similarity surface indicate that most HUCs fall within a reasonable extrapolation distance from RBT training locations.

In evaluating the ensemble RBT occurrence model, we identified Land Surface Temperature, Surface Water Occurrence, and Heat Insolation Load as key predictive indices explaining RBT presence and absence (Figure 4). The model results also showed a favorable 30-fold cross validation accuracy score of 0.87. Surprisingly, Gross Primary Productivity did not show up as a top predictor of RBT occurrence, even though it was identified as a key predictor of RBT hybridization. Choropleth maps showed spatial patterns that agreed with known RBT occurrence records within the study area and reveal a strong tendency to predict high RBT relative suitability in main-stem rivers (Figure 5). In particular, the ensemble model predicted high relative suitability in the North Fork of the Flathead River basin and in the upper Flathead River system for both the first and second decade. For a comparison of the component classifier predictions, see the Supplementary Material S3. The predicted RBT occurrences showed relatively small changes between the first and second decades. Although most predicted suitability differences were negligible, the ensemble model predicted a large degree of decreasing RBT suitability in the Salish Mountains and Lewis Range, with increased suitability in the northern Mission mountains and East Glacier Park regions (Figure 6). The multivariate environmental similarity surface map shows that most HUCs fall within reasonable extrapolation distance from training locations (Figure 7).

**FIGURE 8:**
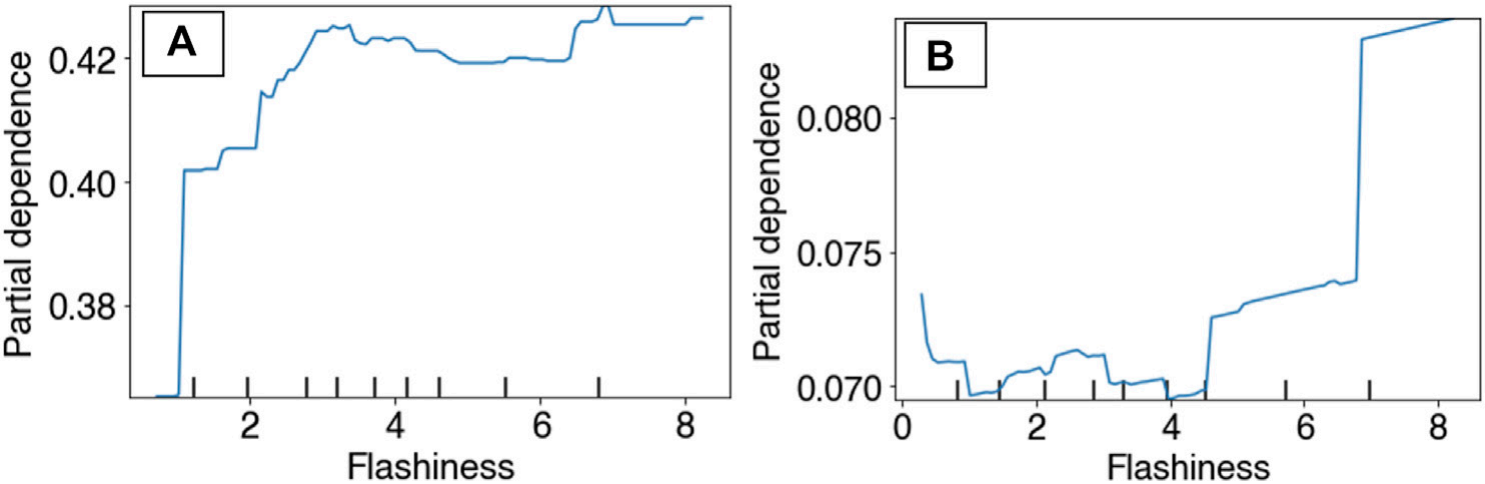
Partial dependency plots for surface water flashiness in both the RBT occurrence ensemble **(A)** and the hybridization ensemble **(B)** models.

**FIGURE 9:**
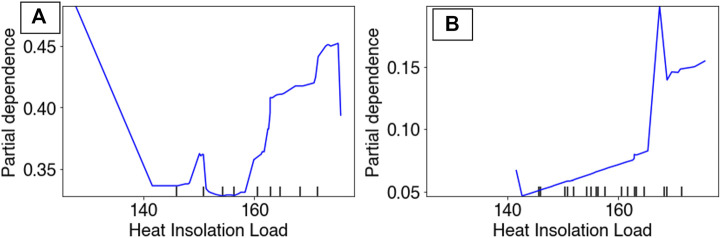
Partial dependency plots for Heat Insolation Load in both the RBT occurrence ensemble **(A)** and the hybridization ensemble **(B)** models.

Partial Dependency Plots (PDP) for the RBT occurrence and hybridization models revealed differing model performances relative to the top predictors, although the PDPs for the RBT occurrence model are more reliable because this model revealed more realistic spatial patterns of habitat suitability (Figure 3). For example, the occurrence PDP for flashiness predicted the highest suitability relative to (unitless) flashiness values of 3, whereas the hybridization PDP for flashiness predicted the highest hybridization levels at 7 (Figure 8). The PDPs for both Land Surface Temperature and Surface Water Occurrence showed similar performance between models, and both models showed increasing suitability at temperatures below 34°C. Although both ensemble models identified Heat Insolation Load as a top predictor, the shape of this PDP differed substantially for both models (Figure 9).

## Discussion

We present a streamlined workflow that can be used for identifying top predictors of species occurrence and evaluating areas of high risk for invasion and establishment of IS in freshwater ecosystems. This case study allowed us to identify strengths, pitfalls, and opportunities for refinement of this workflow. We attained high cross-validation accuracy and identified key environmental predictors. Model performance relative to the top predictors reinforced known assumptions about RBT distributional requirements in the case of the occurrence model.

We place the utility of this methodology squarely in the realm of prediction-first objectives, to be used in tandem with other management tools. Our methodology provides pivotal advancement towards integrating research insights between managers, stakeholders, and decision makers, a crucial step towards proactive IS management (Reaser et al., 2020b). The effectiveness and efficiency of this data-driven approach not only permit managers to objectively prioritize “high-risk pathways” (Pyšek et al., 2020), but also enable frequent sharing of maps created from rapidly mobilized occurrence data (Groom et al., 2019). These advantages allow for weighing the costs and benefits of potential management actions at intervals and time scales relevant to managers. As species occurrence data and temporally dynamic environmental information are received, they can be readily mobilized into actionable products using methodologies similar to the current study.

The lack of spatial continuity of RBT hybridization predictions suggests that our workflow was unable to accurately model this process, in part due to a non-random field sampling effort. Understandably, sampling protocols prioritized streams where there was concern that RBT were hybridizing with native WCT, resulting in an overrepresentation of recent hybrids that may have skewed the distribution of hybridization training data or at least underrepresented hybridization values in the 40–70% range. It remains unclear whether the unreliable model performance was due to the weaknesses of the training information or the difficulty in representing this process from remotely sensed data products. Indeed, modeling hybridization may not be possible without incorporating a clear dispersal mechanism in the model. In fact, RBT hybridization appears to be driven more by propagule pressure than environmental conditions (Muhlfeld et al., 2017). Thus, results of the hybridization model must be interpreted cautiously—unless stated explicitly, the remainder of this discussion addresses the RBT occurrence model.

Correlative approaches to evaluating relative habitat suitability are well suited to the EDRR framework, although the tree-based models (both hybridization and occurrence) performed relatively well without additional tuning steps and could be better suited to EDRR. Reaser et al. (2020a) define EDRR as a “guiding principle for minimizing the effects of invasive species in an expedited, yet effective and cost-efficient manner.” Here, we demonstrate that readily-available data products and empirical machine learning models can facilitate these foundational principles and specifically address the target analysis portion of the EDRR paradigm. Due to their flexibility and swiftness without the need of tuning procedures, tree-based ML models are especially suited to this stage, which is characterized by intensive surveys and proactive biosurveillance to detect the presence of IS with limited resources (Ricciardi et al., 2017). This spatial prioritization tool is critical during the early stages of invasion (Carlson et al., 2019), and managers using our workflow could prioritize high suitability areas to maximize the effectiveness and cost-efficiency of field efforts. For example, our occurrence model predicts high RBT suitability in the North Fork of the Flathead River and therefore suggests that monitoring efforts could be focused in that region. In addition, identifying top environmental drivers of RBT occurrence allows for more robust assessments of shifting conditions as observational data products are updated and released.

The fact that LST was still identified as a top predictor in both the hybridization and occurrence models suggests that temperature is an important driver of RBT distributions in this region. In addition, our connectivity metric (Surface Water Occurrence) was identified as another top predictor in the case of the more robust RBT occurrence model. However, the steep topography and dense riparian vegetation of stream ecosystems create a challenge for interpretation. For example, the global surface water extent algorithm does not include water bodies of less than 30 × 30 m, is known to underestimate water occurrence under emergent vegetation, and resolves the effects of terrain shadows via slopes derived from a 30 m DEM (Pekel et al., 2016). Indeed, the diverse vegetation communities and structural heterogeneity of aquatic systems biases the detection capability of this product towards open areas and larger stream orders. Similarly, although the LST product has been linked to stream temperature at the basin or reach level, the connection is less clear in smaller streams, particularly in those with mixed inputs (McNyset et al., 2015). Aggregating at a HUC scale mitigates some adverse effects but does not preclude all issues of scale mismatch. Still, given the above caveats, a cautious interpretation of model performance against such predictors is insightful.

**FIGURE 10:**
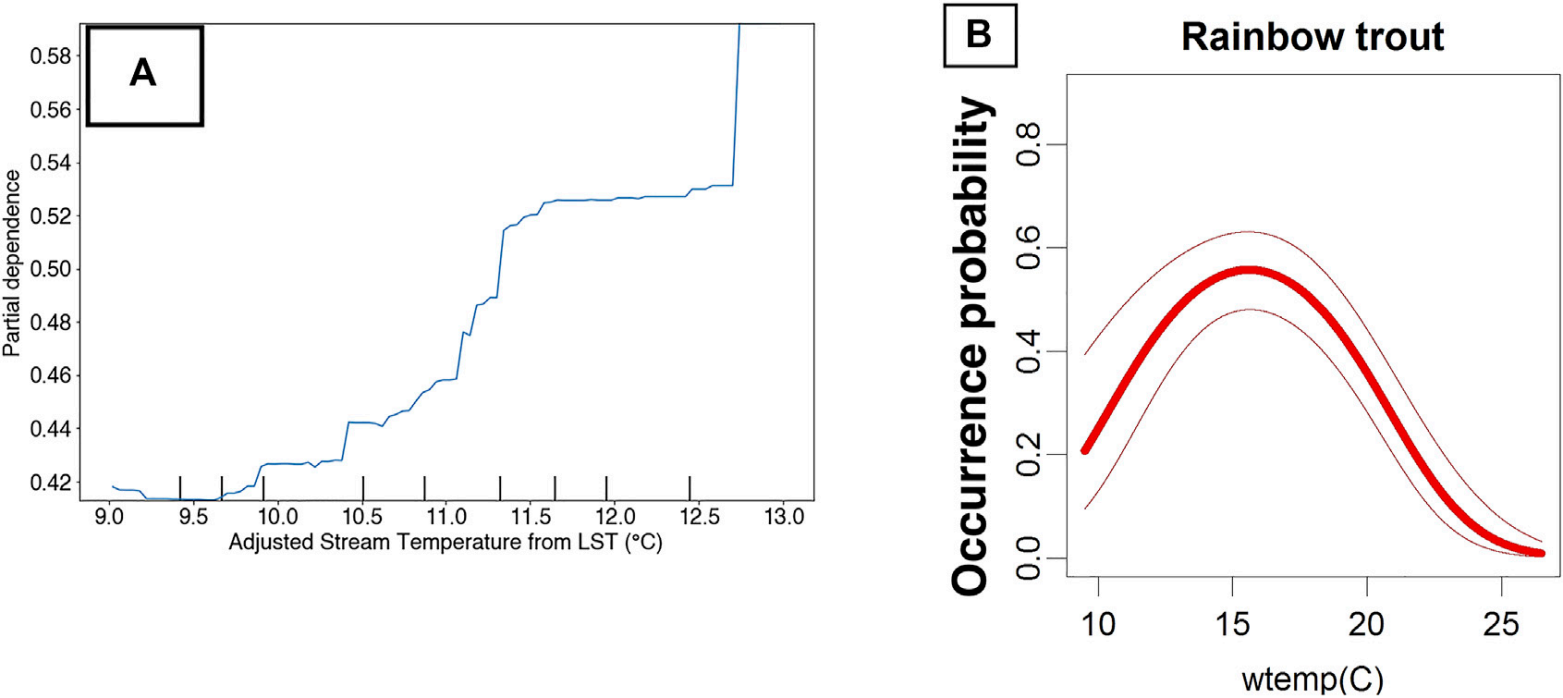
Partial dependency plot showing RBT occurrence model performance against stream-temperature adjusted Land Surface temperature in the Flathead River basin **(A)** versus predicted water temperature (wtemp) niche requirements of RBT **(B)** from Wenger et al. (2011).

**FIGURE 11:**
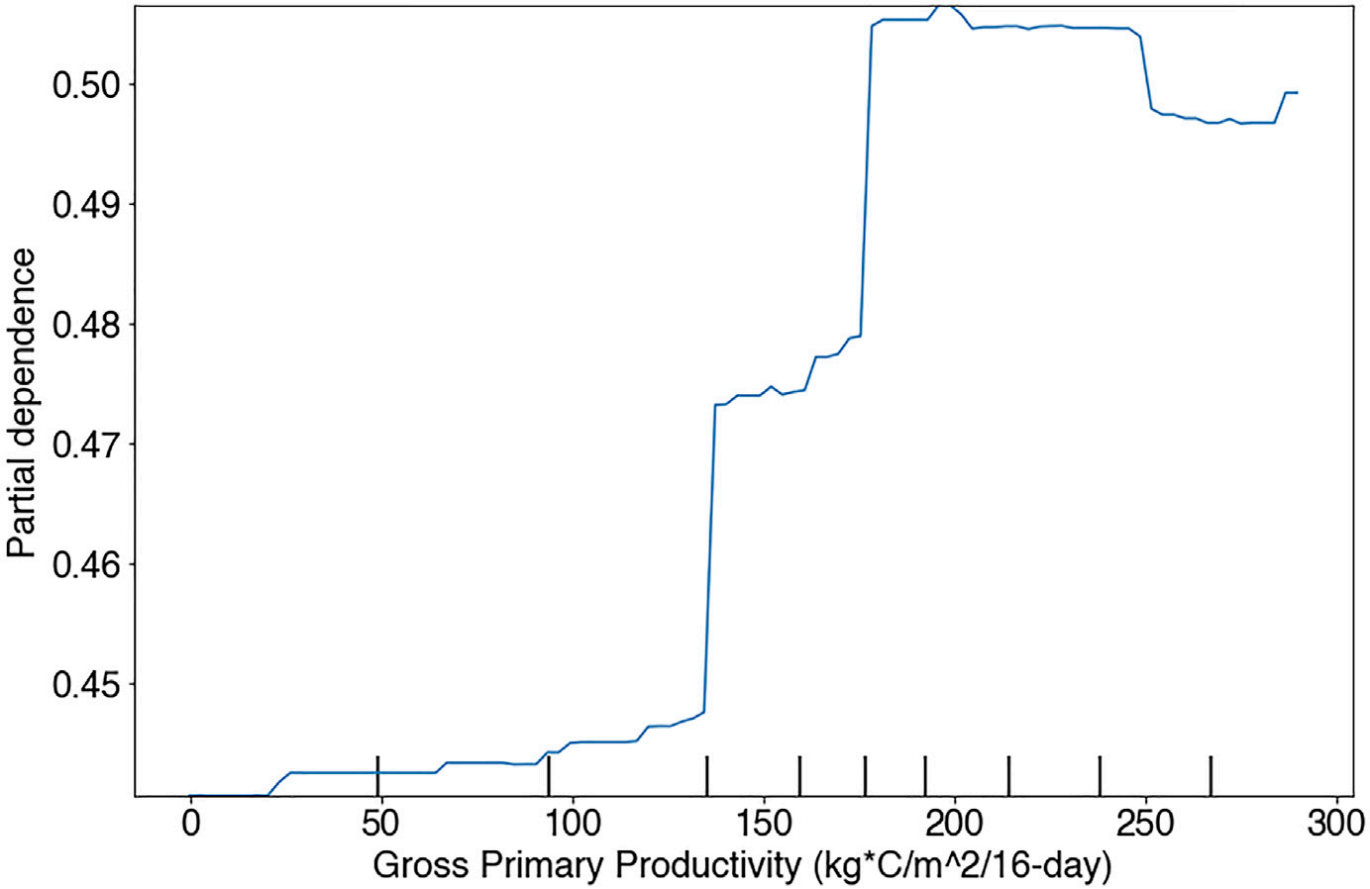
Partial dependency plot showing RBT occurrence model performance against Gross Primary Productivity in the Flathead basin study region.

Specifically, the sign and magnitude of PDPs (i.e., Partial Dependency Plots) relative to proximal predictors of known niche requirements of RBT can be interrogated for realism. For example, the occurrence model predicts increasing relative suitability with increasing LST. Previous research has revealed that LST and stream temperature follow a linear relationship at roughly a 3:1 slope in the Columbia River Basin (McNyset et al., 2015). After adjusting for this relationship, the occurrence model predicts increasing suitability at our highest observed stream temperature of 13°C, and Wenger et al. (2011) found that RBT have optimal temperatures at 16°C (Figure 10). However, not all PDPs showed realistic model performance. For example, the PDP for GPP showed an unrealistic dip at 250 kg C/m^2^/16-days (Figure 11).

Interrogating relatively low-importance model predictors can also be valuable. There were a few such products whose lack of explanatory power can be attributed to temporal lag effects, scale mismatch, or model uncertainty. For example, EVI has been used as a proxy for submerged aquatic vegetation in open water systems (Massicotte et al., 2015), although the connection to species richness in streams is less clear (Vieira et al., 2015). Thus, EVI may not translate to ecologically relevant conditions for RBT within the spatial and temporal scale of our study. Similarly, a terrestrial GPP metric was the most important variable in predicting global-scale species richness of freshwater fish (Pelayo-Villamil et al., 2015) and is correlated with fish production in lakes (Downing et al., 1990). However, our analysis did not reveal GPP as an important predictor for RBT.

Given that GPP represents terrestrial carbon available to primary producers (Robinson et al., 2018) and provides the basis for energy flows supporting aquatic food webs (Welti et al., 2017), it may not drive the higher-level trophic response of stream vertebrates until after a lagging period. In addition, the NLDAS seasonal precipitation metrics did not show up as top predictors, even though RBT are known to be sensitive to peak flow events (Fausch et al., 2001). One possible explanation is the geographic bias present in such spatially interpolated climatic data. Indeed, an examination of the weather stations used in the NLDAS product reveals that geographic coverage of the regional weather station network may be too sparse to fully represent the climate distribution imposed from relatively complex terrain and orographic effects in the Pacific Northwest (Mo et al., 2012). Thus, we recommend the use of landscape scale RS products because of their spatial contiguity. Lastly, although the seasonal additive aggregate model inputs (i.e., Spring Total Precipitation, Summer Total Precipitation) may have captured the magnitude of peak flow events, these aggregates did not inform the timing and duration of flow. More work is needed to integrate the temporal variability of dynamic data products into our workflow.

Our workflow compromises interpretability for speed, accuracy, and efficiency. Top predictors are correlative at best, and without explicitly modeling the dispersal potential of these organisms, our model predicts relative habitat suitability alone. In addition, using temporally composited covariates results in a loss of information relating to the timing and duration of environmental conditions. However, such improvements would compromise the speed and agility strengths of this workflow. As the rate of new biological invasions shows no sign of slowing (Seebens et al., 2017), early detection and rapid response is becoming more vital to prevent irreversible ecological damage and massive economic costs to societies. New technological integrations are needed to facilitate aquatic IS detection and promote proactive management. We present and test one such generalizable workflow for integrating occurrence information with readily available data products to generate spatiotemporally explicit habitat suitability (i.e., risk) maps. While this application case study was for RBT, the underlying models and workflow can be readily extended to other aquatic and terrestrial species.

Given further testing and validation, this workflow could be expanded in its geographic and taxonomic breadth by exploiting web-hosted databases of species occurrence data (e.g. GBIF, www.gbif.org; USGS NAS, http://nas.er.usgs.gov). Future considerations include accounting for sampling bias, integrating presence-only rather than presence-absence datasets, and working toward fully automating the data acquisition and preprocessing steps. The advancement of data sharing capabilities in ecological sciences, born out of the field’s recent rebirth as a big-data science, has enabled robust methodologies and automated pipelines that can produce actionable insight based on continuous occurrence and environmental data streams. Leveraging workflows such as this provide a major step in the way of integrating these data with management action at broad spatial and ecological scales.

## Data Availability

Environmental data were publicly available and were accessed using Google Earth Engine. Some occurrence data analyzed in this study were publicly available. This data can be found here: http://nas.er.usgs.gov. For the remainder of the occurrence data, please contact corresponding author.
