# Supplementary material for: Testing a Generalizable Machine Learning Workflow for Aquatic Invasive Species on Rainbow Trout (Oncorhynchus mykiss) in Northwest Montana
Source: Front Big Data. 2021 Oct 18;4:734990. doi: 10.3389/fdata.2021.734990 (PMC8558495; doi:10.3389/fdata.2021.734990)
Supplement: Supplementary file 1 [file DataSheet1.docx]

Supplementary Material

**S1) Detailed quality control filtering actions**

| **Data** **Product** | **Quality Control (QC) Flag Description** | **Quality Filtering Action** |
| --- | --- | --- |
| Land Surface Temperature | Bits 0 and 1:   - 0: Pixel produced, good quality, not necessary to examine more detailed QA - 1: Pixel produced, unreliable or unquantifiable quality, recommend examination of more detailed QA - 2: Pixel not produced due to cloud effects - 3: Pixel not produced primarily due to reasons other than cloud (such as ocean pixel, poor input data)   *(Etc)* | Only used pixels where bits 0 and 1 equal 0 |
| Gross Primary Productivity | Value 10: Clear not smoothed  Value 11: Clear smoothed  Value 20: Snow or water not smoothed  Value 21: Snow or water smoothed  Value 30: Climatology not smoothed  Value 31: Climatology smoothed  Value 40: Gap filled not smoothed  Value 41: Gap filled smooth | Only used pixels where QC band equaled 10 or 11 |
| Enhanced Vegetation Index | Bits 0 and 1:   - 0: Pixel produced with good quality - 1: Pixel produced, but check other QA - 2: Pixel produced, but most probably cloudy - 3: Pixel not produced due to other reasons than clouds   *(Etc)* | Only used pixels where bits 0 and 1 equal 0 |
| Percent Tree Cover | Bit 0: State of input layers DOY 065-097   - 0: Clear - 1: Bad   Bit 1: State of input layers DOY 113-145   - 0: Clear - 1: Bad   Bit 2: State of input layers DOY 161-193   - 0: Clear - 1: Bad   Bit 3: State of input layers DOY 209-241   - 0: Clear - 1: Bad   Bit 4: State of input layers DOY 257-289   - 0: Clear - 1: Bad   Bit 5: State of input layers DOY 305-337   - 0: Clear - 1: Bad   Bit 6: State of input layers DOY 353-017   - 0: Clear - 1: Bad   Bit 7: State of input layers DOY 033-045   - 0: Clear - 1: Bad | Only used “Clear” pixels for all bits |

Table S1. Detailed description of pre-published quality filtering heuristic rules and our stringent quality masking procedures.

# S2) Description of Machine Learning model implementation

Logistic Regression functions within the maximum likelihood framework by performing gradient descent on the error surface characterized by the difference between observed and predicted suitability (Cramer 2003). A classification tree is built by splitting the input data into successive “leaves” to minimize the g*ini impurity* between consecutive layers of the canopy (Quinlan 1986). Random forests are trained using a series of such classification trees on independently bootstrapped samples (Breiman 2001). Boosted Regression Trees are similar to random forests, but instead recursively build each classification tree using the remaining errors left by previous, intentionally “shallow” trees rather than independently bootstrapped samples of the same data used to train stronger learners (Mishina et al., 2015). XGBoost is a more generalizable form of Boosted Regression Trees that incorporates regularization and a more accurate gradient descent algorithm (Chen and Guestrin 2016). Neural networks are trained using a gradient descent algorithm that minimizes the error between observations and predictions (McCulloch and Pitts 1943).

**S3)**

**
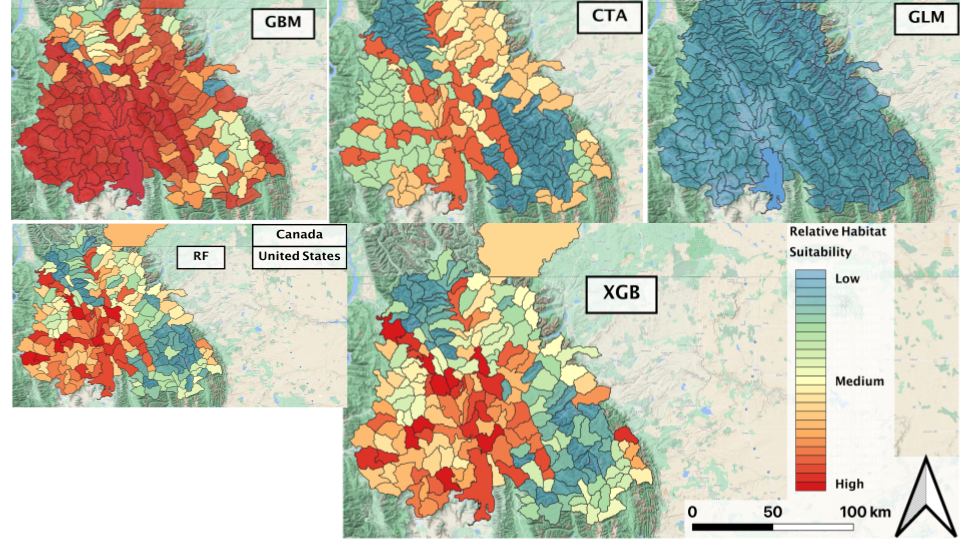
**

Figure S3. Comparison of each component classifier. Bagging and boosting classifiers (i.e. GBM, RF, and XGB) all predict higher habitat suitability in the southwest portion of the study area, an area which was shown to be outside of the training envelope (Figure 7). The ANN was not included due to space. However, it shows the exact opposite of the GLM (i.e. it predicts high suitability for all regions of the study area).
